# Enhancement of Esterification Reaction Rates in Solvent-Free Aerosol Droplets

**DOI:** 10.1021/jacs.5c18218

**Published:** 2026-03-20

**Authors:** Joshua Harrison, Aleksandra Marsh, Rachael E. H. Miles, Allen E. Haddrell, Bryan R. Bzdek, Jonathan P. Reid

**Affiliations:** School of Chemistry, 1980 University of Bristol, Cantock’s Close, Bristol, BS8 1TS, United Kingdom

## Abstract

Aerosols are appealing vessels for accelerating chemical reactions owing to their high surface area-to-volume ratios and capacity to access supersaturated solute states. Although many studies demonstrate enhanced reaction rates in charged droplets using electrospray ionization-mass spectrometry, such studies have poor control over experimental parameters including droplet size, water content, and imparted charge and generally cannot explore the reaction’s reversibility. Here, an esterification reaction between a dicarboxylic acid and low-volatility alcohol (that in bulk synthesis requires reflux at 160 °C for ∼15 h in nonaqueous solvent) is investigated in aqueous aerosol droplets spanning picoliter to sub-femtoliter volumes. Individual picoliter droplets generated without applied external voltage were confined within an Aerosol Optical Tweezers instrument, and Raman spectroscopy was used to resolve chemical change in the droplets. Flow tube experiments on sub-femtoliter aerosol droplets were conducted using both online aerosol mass spectrometry and off-line NMR spectroscopy. Across both droplet volume scales, the esterification was facile (<400 s in picoliter droplets, <2 s in sub-femtoliter droplets) upon droplet dehydration at room temperature. We demonstrate that both the forward and reverse reaction are vastly accelerated in aerosol compared to macroscopic solution. The unique aspects of this study include rigorous control over a water activity range spanning dilute to solvent-free conditions, investigation across a broad droplet volume range, application of multiple analytical techniques to confirm independently accelerated reactivity, and exploration of the reaction’s reversibility. Combined, this study demonstrates how aerosols can drive chemistry in accordance with Le Chatelier’s principle on both the micro- and nanoscales.

## Graphical abstract

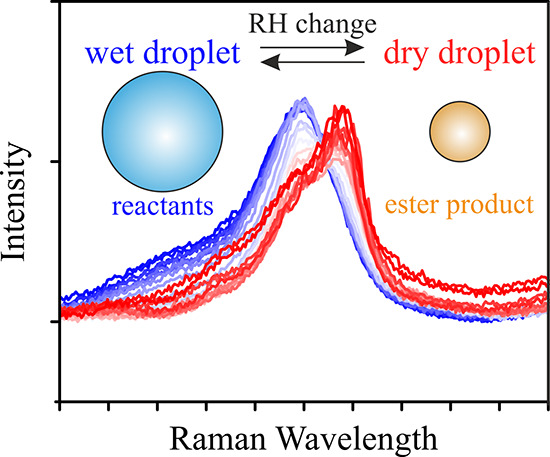

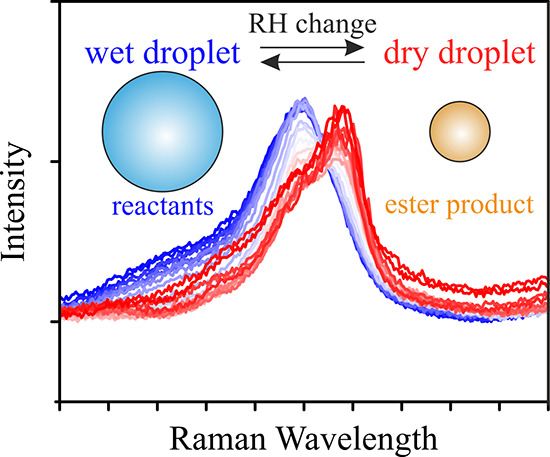

## Introduction

Aerosol droplets provide a unique environment for exploring and performing chemical reactions. The large surface area-to-volume ratios typical of most aerosol dispersions lead to facile coupling between gas phase reagents and the condensed phase reaction volume, and they amplify the role for surface-mediated chemistry when compared with the bulk phase.^1–3^ The unique properties of a water–air surface can lead to control over molecular orientations, enhancements in concentrations of solutes, and atypical acidity/basicity.^4,5^ In addition, the absence of a solid substrate and, thus, sites for heterogeneous nucleation can allow droplets to access metastable supersaturated solution states with extremely high solute (reactant) concentrations. Typical aerosol droplet sizes can span 10 nm to 10 μm, corresponding to a wide range in volume (<10^–20^ to >10^–11^ dm^3^) and clearly representing examples of reaction chemistry in confined volumes.

Chemical transformations in aerosols are widespread.^6–9^ Organic species in ambient atmospheric aerosol generally progress by a combination of functionalization, fragmentation, and oligomerization reactions, leading to changes in average oxidation state, distributions in molecular mass, and variations in component volatility.^10,11^ Reactions are largely assumed to progress toward products in a manner consistent with bulk phase chemistry, benefiting from a conventional understanding of reaction mechanisms, thermodynamics, and kinetics. However, some transformations occurring in aerosol proceed because of the unique environment aerosols provide, particularly due to dehydration and increasing solute concentration.^12–28^ For example, dehydration-driven aqueous phase chemistry of small species can lead to the formation of oligomers and light-absorbing compounds.^29–34^ Further, aerosols exposed to variable temperatures and humidities at supersaturated reactant concentrations have been proposed as prebiotic chemical reactors, which could have played a role in the origin of life.^35,36^ Droplets provide a rich environment in which to study reaction kinetics and mechanisms in confined environments,^2,3,37–39^ with the possible long-term opportunity to exploit aerosols as a platform for performing chemical transformations under unconventional conditions, benefiting from their unique interactions with light and facile coupling to the gas phase.

Numerous studies have explored enhancements of reaction rates in evaporating charged droplets but at unspecified or uncontrolled solvent activity. Droplet formation and monitoring of the reaction progression are achieved using variations of electrospray ionization mass spectrometry (ESI-MS).^12–16,26,40^ For example, Lee et al. observed changes to molecular mass on the fusion of two highly charged reactant droplets at variable distances/times following coalescence. For the reaction between 2,6-dichloro-phenolindophenol and ascorbic acid, they reported an increase in rate by 6 orders of magnitude in the droplet phase when compared with similar reactions conducted in the bulk and identified intermediate and product species by MS.^18^ Using the same approach, the Pomeranz–Fritsch synthesis of isoquinoline and the Friedländer and Combes syntheses of substituted quinolines were explored on millisecond time scales in charged microdroplets, demonstrating an enhancement by 6 orders of magnitude when compared to rates in the bulk phase.^24^ Chen and Williams used theta-glass electrospray emitters to mix solutions in aqueous aerosol droplets, analyzing reaction rate enhancement in evaporating droplets and proving the significance of reactant concentration to accelerated kinetics upon solvent evaporation.^41^ Bain and co-workers drove the Hantzsch synthesis through generating droplets by electrospray. In droplets, the reaction took place on a time scale of milliseconds (yield >90%) without the need for the phenylboronic acid catalyst. In the bulk phase, the reaction proceeded with a 96% yield but required over 16 h of reflux with phenylboronic acid as a catalyst; without the use of the phenylboronic acid catalyst and reflux, only a 37% yield was obtained over 64 h.^13^

The above studies considered charged microdroplets dispersed in the gaseous phase. However, accelerated chemistry is also observed in the absence of substantial net charge on the droplet, again with uncertainty over the exact solute concentrations and solvent activities. For instance, uncharged Leidenfrost microdroplets also showed enhancement of reaction rates, with suppression of enhancement on addition of surfactant to the droplet, demonstrating the possible importance of the surface in mediating enhancements in chemical reaction rates.^15^ Further, Fallah-Araghi et al. used an uncharged emulsified colloidal system dispersed in a microfluidic device to investigate rate enhancements in confined environments. The concentration of a fluorescent imine product was monitored following mixing of a nonfluorescent amine and a weakly fluorescent aldehyde in aqueous droplets of 8–34 μm radius. Results suggested that the product yield was inversely proportional to the droplet radius, and imine formation was 45 times faster in 8 μm droplets when compared with bulk reaction rates.^42^

Condensation reactions, similar to the ones described in this work, have been observed to occur at accelerated reaction rates, particularly in the field of abiotic synthesis. While comparing the effect of charge via monitoring reaction kinetics with charged and uncharged droplets, again the solute concentrations and solvent activities are largely unreported or studied only over limited ranges.^43–45^ The condensation reaction whereby two pyruvic acid (PA) molecules react to produce zymonic acid (ZA) has been extensively studied, providing an opportunity to explore the dependence of reaction rate on droplet size, over a limited range in relative humidity (RH) and with a change in temperature. Wilson and co-workers have concluded from an aerosol flow reactor coupled to MS that the reaction rate enhancement arises exclusively from the reaction occurring at the aerosol surface.^46^ They also measured the rate for the same reaction at interfaces in oil–water emulsions and found a higher degree of enhancement than that at the air–water surface. However, a lower propensity of PA for the oil–aqueous relative to air–water surface leads to a slower overall rate of product production.^47^ In measurements on large sessile droplets (>80 μm radius), Grassian, Dutcher, and co-workers found that the droplet size-dependent reaction rates for the PA condensation reaction can best be explained by the inclusion of an autocatalysis step where the ZA product catalyzes the condensation reaction.^48,49^ Their measurements explored reaction rates at RH values spanning 65–95%. They also highlighted the complexity of modeling the reaction rates for systems where concentrations of reactants and products are continuously changing, water content is changing due to any imbalance of water activity with the surrounding RH and differences in hygroscopicity between the reactants and products, and reactants and products can partition into the vapor phase.^50^ Indeed, they concluded from their measurements that the evaporation of PA is a necessary requirement for the reaction rate to be enhanced at the droplet surface. Grassian and co-workers recently extended their work to investigate reactions of C_8_ to C_16_ carboxylic acids with C_1_ to C_3_ alcohols in microdroplet sprays, applying optical photothermal infrared spectroscopy for the analysis of deposited droplets.^51^ Moreover, the reaction of carbonyl functionalities with Girard’s T reagent has been shown to be accelerated during desorption ESI studies,^26,52^ and controlled aerosol flow tube experiments estimate this reaction is accelerated by ∼4 orders of magnitude in aerosol relative to bulk solution, with the reaction preferentially occurring at the aerosol–air interface.^53^ As an indication of the potential for synthesizing organic molecules in droplets on a larger scale, Pena et al. demonstrated that condensation reactions can be facilitated by using spray-drying, significantly reducing reaction times without compromising product yields.^54^ Further, they demonstrate the high-yield production of paracetamol.

Here, we report on a series of esterification condensation reactions between a low-volatility alcohol and a dicarboxylic acid across picoliter (∼10 μm diameter) to sub-femtoliter (∼100 nm diameter) droplet volumes, with the picoliter droplet experiments proceeding in single-particle optical traps and the sub-femtoliter experiments in an aerosol flow reactor. Aerosol composition in these two size regimes is measured by complementary tools, including microdroplet Raman spectroscopy for picoliter droplets and online MS and offline NMR for sub-femtoliter droplets. The application of *in situ* Raman spectroscopy to study individual picoliter droplets allows interrogation of both product formation and reactant loss in the same droplet. Recent work has demonstrated the mechanistic insights into reaction rate enhancements that can be achieved by studying chemistry in single suspended droplets.^55^ Droplets were generated without applied external voltage, allowing investigations into increased reaction rates due to water (i.e., solvent) loss and increased reactant concentration. Uniquely, the reaction is performed in solvent-free droplets by drying the aerosol to 0% RH (taking advantage of slow nucleation kinetics for crystallization), with droplets composed solely of the alcohol and carboyxlic acid reactants and without any water. Finally, we observe that esterification is reversible across both droplet volume ranges upon sequentially decreasing and increasing the RH, indicating that both the forward and backward reactions are catalyzed in aerosol droplets.

## Experimental Section

We explored aerosol-phase reactions across multiple time scales (1–1000s of s) and droplet length scales using single levitated picoliter droplets (∼10 μm diameter) and plumes of sub-femtoliter aerosol droplets (∼100 nm diameter), with droplet compositions measured using Raman spectroscopy (levitated droplets), NMR spectroscopy (collected plumes of sub-femtoliter droplets), and online MS (flowing plumes of sub-femtoliter droplets).

### The Reaction: Esterification between a Dicarboyxlic Acid and Carbitol

The esterification reaction studied is shown in Scheme 1. Aqueous solutions of glutaric acid (abbreviated as GA herein and labeled A in Scheme 1) and Carbitol (B) with varying molar ratios have been studied, spanning from binary aqueous solutions of GA or Carbitol alone to mixtures that include stoichiometries of 2:1 and 3:1 Carbitol:GA to improve formation of the monoester (C) or diester (D). GA is part of a homologous series of dicarboxylic acids; succinic acid (SA) has one less carbon atom and was also studied. Kokyu Alcohol Kogyo Co. Ltd. patented the preparation of diesters with the generic formula shown in Scheme 2. The diesters have good solubility in both water and oil, have low viscosity, and are used in cosmetics (shampoos, makeup products) and in lubricants, paints, and inks.^56^ The preparation of dicarbitol succinate is described in detail in the patent. SA and Carbitol are combined with a *p*-toluenesulfonic acid catalyst and toluene solvent and refluxed at 160 °C under nitrogen for 15 h. Sodium carbonate decahydrate is added for a further hour at 120 °C. Different dicarboxylic acid reactants require variable reflux times which range from 14 to 18 h. The patent also provides IR data for the dicarbitol succinate product and reports the ester carbonyl peak at 1732.98 cm^–1^, which is useful to assess any changes in carbonyl wavelengths detected in Raman spectra.

**Scheme 1:**
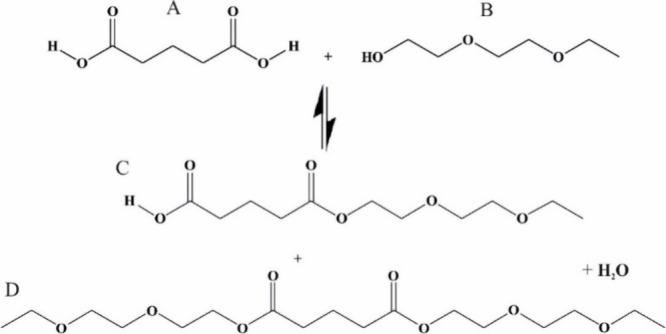
Possible Reaction Products in the Reaction between GA (A) and Carbitol (B)

**Scheme 2:**
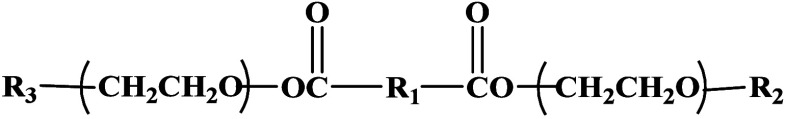
Generic Structures Covered by the Kokyu Alcohol Kogyo Co. Ltd Patent

### Aerosol Optical Tweezers (10–20 μm Diameter Droplets)

The esterification reaction was studied in picoliter volume droplets using Aerosol Optical Tweezers (AOT). The AOT setup has been described in detail in previous publications.^57^ Droplets are captured from a nebulized flow (mass median diameter of 4–5 μm) at high RH/water activity (typically 80%) in an optical gradient force trap (optical tweezers) established by focusing a 532 nm laser beam through a high numerical aperture microscope objective. At the initial water activity in the trapping cell, the solute concentrations are ∼1 M. Mixing dry and humidified nitrogen gas flows into the trapping cell provides control over RH. The reaction is initiated when the RH is reduced to close to 0% RH. The Raman spectrum provides a highly accurate method for determining droplet radius and refractive index (RI, <±0.05% on both measurements),^58^ as well as for examining Raman signatures of reactants and products. Because the droplet remains spherical even at low RH, the appearance of whispering gallery modes (which permit retrieval of the droplet radius and refractive index) is sustained across the entire experiment.

### Aerosol Flow Reactor (∼100 nm Diameter Particles)

Figure 1 shows a schematic of the aerosol flow reactor. This experiment has the aim of reproducing the reaction conditions achieved in the AOT with Carbitol/GA solutions but for an ensemble of sub-femtoliter droplets. The products are characterized by postanalysis of a collected sample using ^1^H NMR. A flow of aerosol (median diameter ∼ 100 nm) is generated from a TSI atomizer with a dry nitrogen gas flow (2 L·min^–1^). The atomizer solution contains an aqueous solution of Carbitol and GA in a 3:1 ratio (6.5 M Carbitol, 2.2 M GA). The aerosol is first dried by passing the flow through a pair of Nafion driers, ensuring the RH is sufficiently low (measured to be <10%) for the reaction conditions to be equivalent to those in the AOT. Diethyl ether was chosen for use as a collection solvent for the aerosol, as it has a relatively low boiling point and is, thus, easy to separate from the product with gentle heating. Dried aerosol was left to flow into the diethyl ether bubbler for approximately 45–60 min. Diethyl ether was then collected from both the boat and the bubbler into separate vials, and each vial was gently heated to evaporate the solvent while avoiding water absorption and loss of product. After drying, the formation of a white crystal was observed in the vial containing the bubbler solution, which was subsequently analyzed using ^1^H NMR.

**Figure 1:**
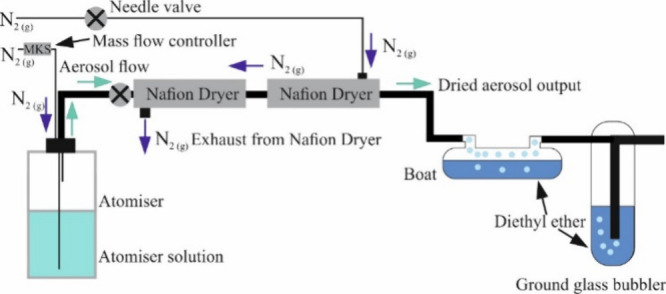
Schematic of the atomizer and Nafion dryer aerosol flow reactor. The aerosol is generated using the atomizer and dried using the Nafion dryer, and the resulting dried aerosol product is collected using a bubbler and boat which are filled with diethyl ether. The boat equilibrates the aerosol to a gas phase of diethyl ether before collecting in the bubbler, minimizing evaporation from the bubbler over the course of the collection period.

### Mass Spectrometry Experiments (∼100 nm Diameter Particles)

A 2:1 molar ratio Carbitol/GA aqueous solution was used for the MS experiments (1 mM Carbitol and 0.5 mM GA). The reaction was studied primarily using Droplet Assisted Ionization-Mass Spectrometry (DAI-MS).^59,60^ In DAI, a plume of aqueous droplets generated without applied external voltage is sampled from ambient pressure into a heated capillary inlet attached to the MS. Ions are generated from the aerodynamic or thermal breakup of the droplets within the MS inlet.^61^ In the aerosol setup used here (and analogous to the flow reactor measurements), droplets were atomized from aqueous solution with an atomizer (TSI 3073), equilibrated (when desired) to a chosen RH in a Nafion dryer, and then delivered to the MS inlet for chemical analysis. A quadrupole time-of-flight mass spectrometer (Waters Synapt XS) was used for MS analysis. To explore the reverse reaction, in a subset of experiments, the RH-conditioned aerosol was sent to a Condensation Growth Chamber (Series 110A spot sampler, Aerosol Devices) after exiting the Nafion dryer and before delivery to the MS. The Condensation Growth Chamber exposes the aerosol to a supersaturated RH, growing the aerosol by water condensation from submicron sizes to ∼4 μm diameter.^62^ In this way, dried aerosol is rehumidified before delivery to the MS.

## Results and Discussion

Across multiple experiments spanning different droplet sizes and reaction time scales, we report here a combination of circumstantial and direct evidence for the formation of the mono- and diester products under low and no solvent reaction conditions.

### Phase Behavior and Volatility of Mixed GA/Carbitol Aqueous Droplets

To explore the properties of mixed component GA/Carbitol aqueous droplets and the chemical transformations that can occur as a particle is dehydrated, we first consider binary organic/aqueous droplets for each of the two reactants in isolation with measurements made using the AOT. A change in the gas phase RH leads to a change in the water activity in the solution phase droplet. An example of the variation in droplet size and RI for a GA droplet with variation in RH and over a period of ∼20,000 s is shown in Figure 2.

**Figure 2:**
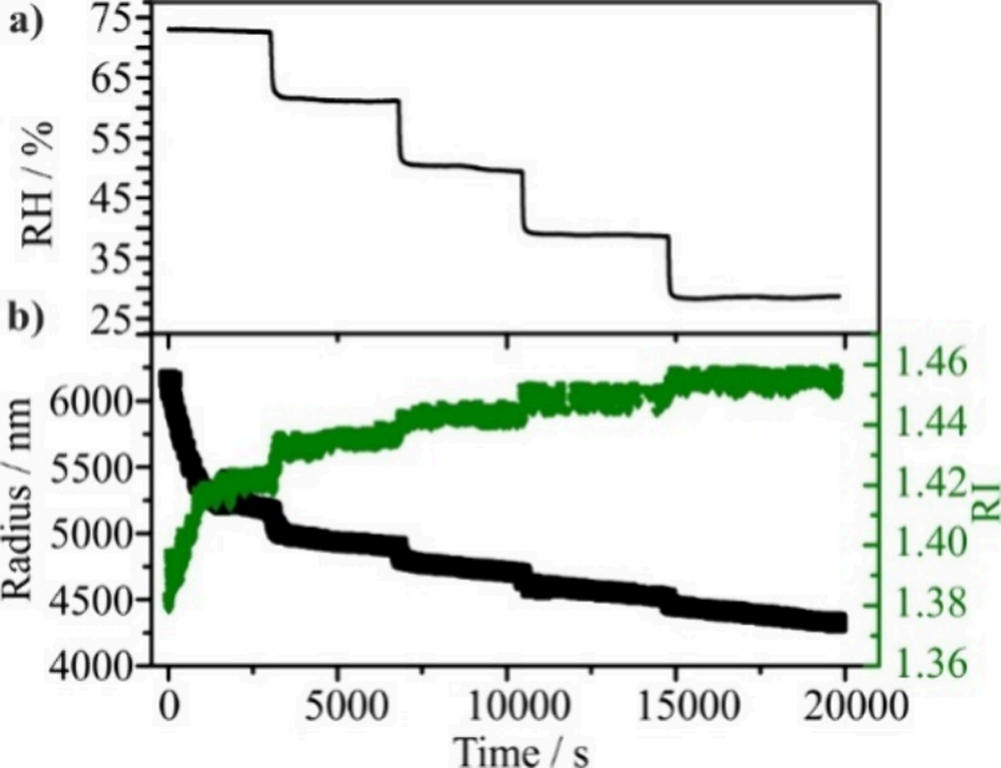
(a) Change in RH and its influence on radius and RI shown in (b) for an aqueous GA droplet (300 g·L^–1^).

As the RH is reduced, the water partitioned to the condensed phase decreases, leading to an increase in the solute concentration for each reduction in RH/water activity. Between each step in RH, a slow decline in size and a rise in the RI to a steady value is apparent, due to the slow evaporation of GA, a semivolatile organic with a vapor pressure of ∼5 × 10^–4^ Pa, for a droplet at constant composition. With a decrease in RH, the rate of evaporation increases as the mole fraction of the evaporating component increases. These observations are consistent with our previous observations of combined measurements of component vapor pressure and hygroscopicities for a binary component aerosol.^30,31^ In this particular experiment, the GA undergoes efflorescence (crystallization) during the constant period of RH at ∼25%, consistent with expectations from past studies.^32^ Analogously, the decline in size with time of aqueous solutions of Carbitol droplets enables determination of its vapor pressure as 10.8 ± 0.13 Pa. Measurements were made using an electrodynamic balance (EDB) approach, as Carbitol evaporates too quickly to be studied using the AOT. (EDB measurements are shown in Figure S1.) The EDB technique employed for the measurements here has been extensively discussed in previous work.^63^

By comparison with the binary-solution droplets, the time dependence in size and RI for a solution droplet in the AOT containing both Carbitol and GA in a 2:1 molar ratio following a sudden reduction in RH is shown in Figure 3(a). Here, the particle is dried to below the efflorescence point of GA at 5% RH and held for more than 5 h. Once the initial drying period at 5% RH is complete (after ∼3000 s) following initial trap loading at 80% RH, the droplet size becomes steady with an RI (∼1.44) that is lower than that of a GA droplet dried to only 25% RH. However, there is no evidence for crystallization, unlike for binary GA/water droplets. The predicted value for the real part of the pure component melt RI of GA is 1.4655 from the molar refraction mixing rule (with an uncertainty of ± 0.002), and the measured value for liquid Carbitol is 1.4272.^64^ The real part of the RI of the combined droplet lies between these two values at ∼1.44, suggesting both components remain. In addition, the evaporation of both organic components is completely suppressed, suggesting a chemical reaction has occurred, again contrasting with the binary component cases.

**Figure 3:**
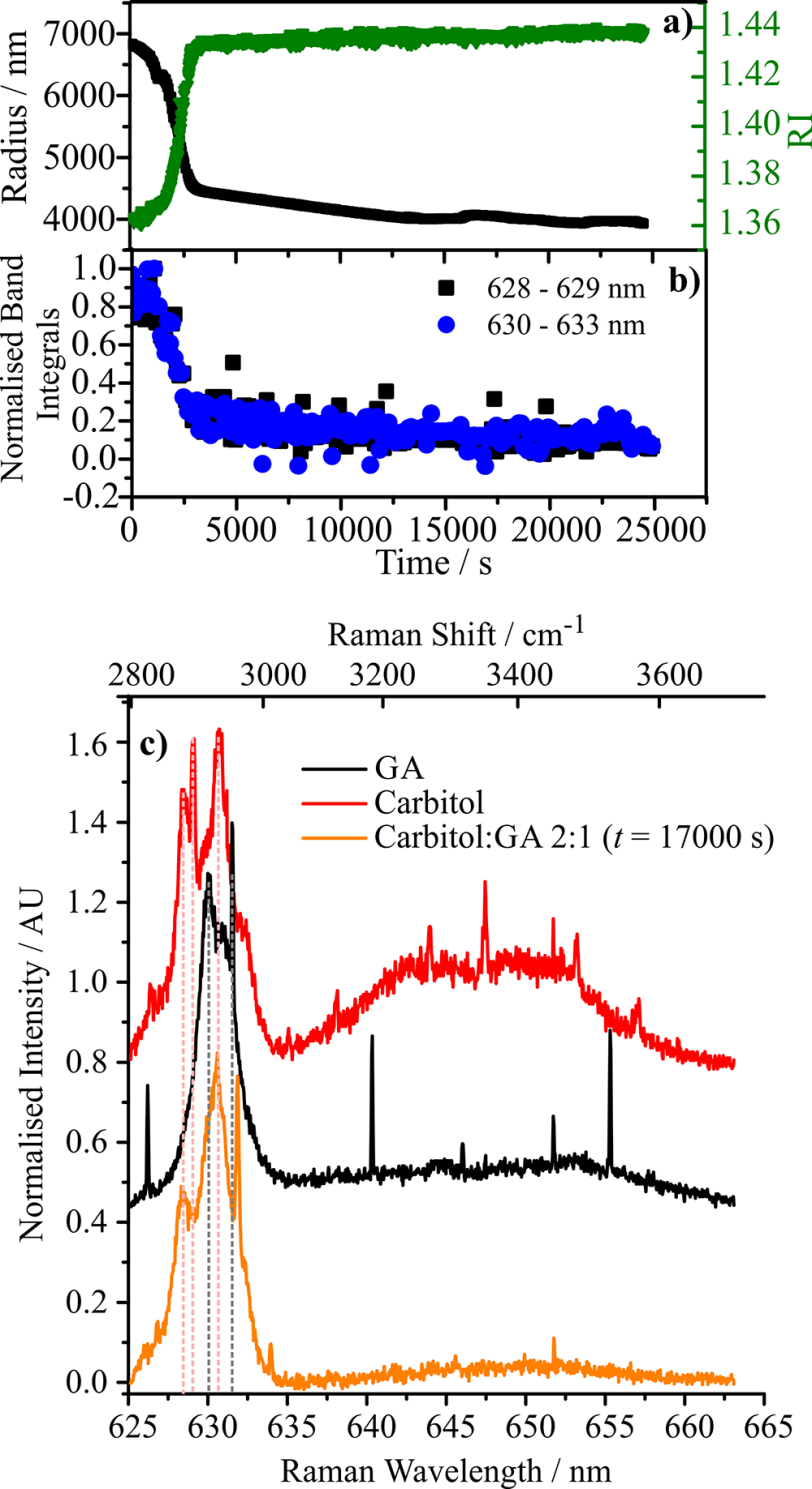
(a) RI (green) and radius (black) for a 2:1 molar ratio mixture of Carbitol and GA as a function of time. After trapping, the RH dropped from 80% to 5%. (b) Normalized band integrals for peaks characteristic of Carbitol (628–629 nm) and GA (630–633 nm). (c) Raman spectra for the droplet experiment presented in (a) and (b) after 17000 s (orange) (note that there is no water obviously present in the orange spectrum) compared with spectra from aerosol solutions containing solely the aqueous reactants GA and Carbitol (at ∼80% RH). In the mixture droplet spectrum, there are similarities in the Raman band shape to the single-component droplets. However, it is clear that the C–H stretching band does have new character with peaks for the pure components (shown by dashed lines) not perfectly aligned to the stretching frequencies from the molecular products. The sharp peaks superimposed on the Raman spectrum arise from whispering gallery modes, owing to the spherical nature of the droplet.

Figure 3(b) shows band intensity measurements at 628–629 nm and 630–633 nm, whereas Figure 3(c) compares spectra of droplets containing only the reactant GA (black), the reactant Carbitol (red), and the reactant mixture at 17,000 s (orange). Figure 3(b) and (c) show a clear and persistent signature of Carbitol “functionality” characteristic of a CH band typically adjacent to a CO bond (628–629 nm), which is unique to Carbitol. This peak is present in both the Carbitol spectra and in the 2:1 mixture after 17,000 s. Hence, the Carbitol functionality is still present in the mixture after prolonged suspension under dry conditions, as shown in Figure 3(b) and (c). Without GA, Carbitol evaporates entirely in <100 s from a droplet at 80% RH. Indeed, at 5% RH, where the mole fraction of GA is larger than at 80% RH, the evaporation rate of Carbitol can be expected to be considerably higher (Raoult’s law) and the lifetime in the droplet even shorter than 100 s.

Combined, the results presented in Figure 3 suggest that GA and Carbitol have undergone a chemical change or are experiencing intermolecular interactions under dry conditions such that the components have considerably lower volatility. The absence of crystallization is not uncommon in aerosol (considerable supersaturations can be achieved in organic aerosol droplets), but this does suggest that the droplet will contain almost exclusively GA and Carbitol with no water and that the concentrations can be assumed to be considerably higher than the initial bulk phase solution. Estimates based on size and initial droplet concentration suggest the concentration of GA may be as high as 5 M. Indeed, the total mass fraction of solute (i.e., reactants) in the particle at <5% RH can be estimated to be higher than 0.9.

### Evidence for Reversible Formation of Esterification Products on Single Droplet Drying

Raman scattering at lower Stokes’ frequencies (<2000 cm^–1^) can provide an invaluable fingerprint of the chemical functionalities present in a sample. In any chemical transformation involving a carbonyl group, including esterification reactions, the change in the carbonyl region (∼1700 cm^–1^) is of most interest. Typically, in our aerosol phase experiments, the spectrograph of the AOT is centered at a wavelength of 650 nm, as this allows for easy fitting of the WGM wavelengths across the relatively broad OH and CH bands (2795–3760 cm^–1^) and the estimation of droplet size and RI. However, the spectrograph must be centered at 575 nm to view the carbonyl region (∼1700 cm^–1^). For droplet data each spectrum is averaged over typically 50–100 acquisitions (1 s each), implicitly averaging over fluctuations of droplet size and thereby minimizing the conflicting influence of whispering gallery modes when inferring changes in spontaneous Raman band intensity ratios. Spectra are then corrected for the background and normalized to the peak intensity maximum. Bulk Raman spectra are typically averaged over ∼10 s and then corrected for the background and normalized to the peak intensity maximum.

Figure 4 shows bulk samples of GA and the 2:1 bulk mixture of Carbitol:GA. Both have the same carbonyl signature indicative of unreacted GA at a Raman shift of 1702.97 cm^–1^ (585 nm), consistent with literature reported values for GA.^39^ Hence, the bulk Carbitol:GA solution simply contained a mixture of the starting materials. Furthermore, over a period of almost two months, the Raman spectra of bulk solutions of 1:1, 2:1, and 3:1 Carbitol:GA mixtures remained unchanged; an example is shown in Figure 4(b) with further bulk measurements available in the SI (Figures S2 and S3) for other compositional ratios.

**Figure 4:**
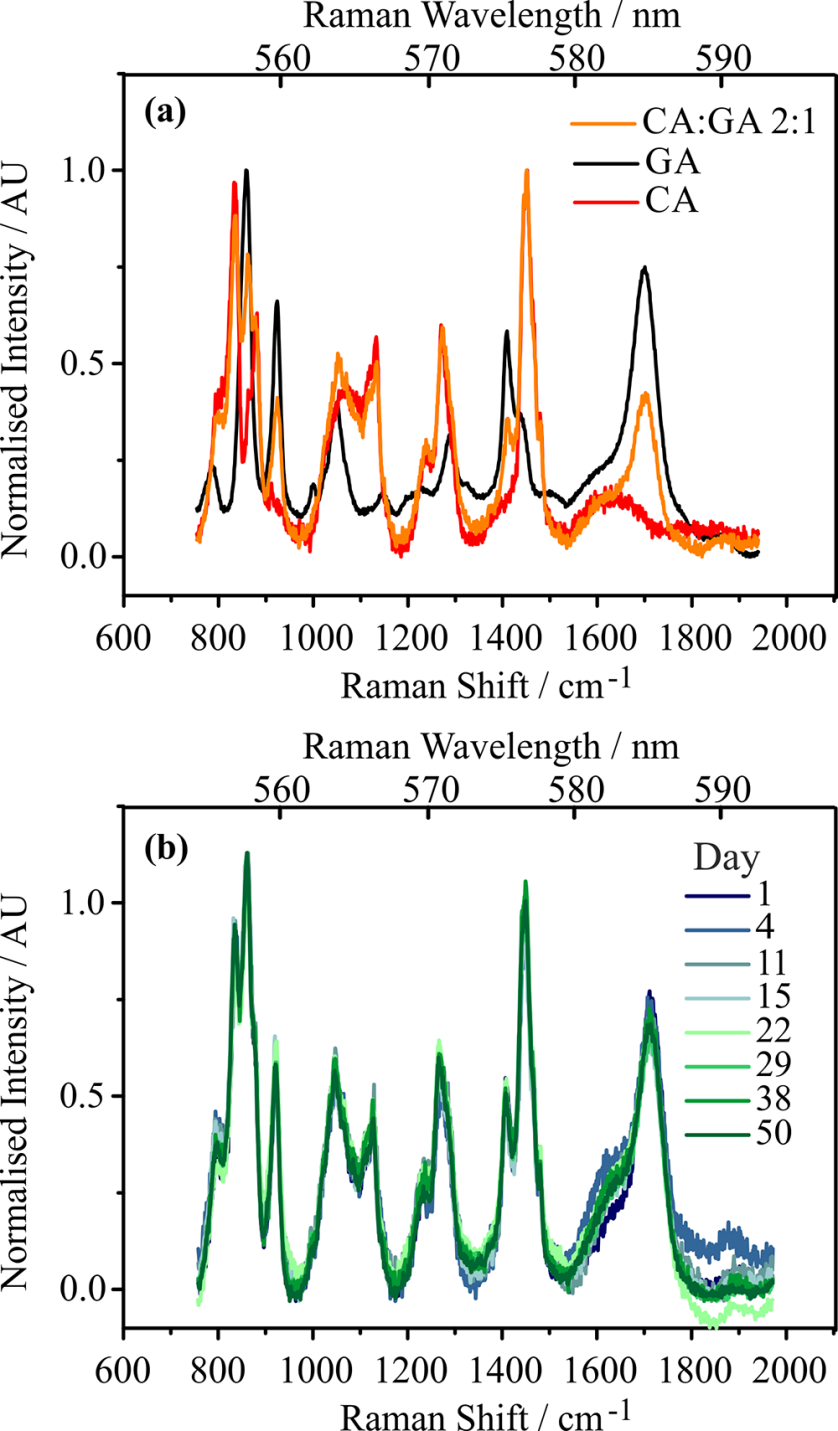
All spectra shown are from bulk Raman samples with the spectrograph centered at 575 nm. (a) An aqueous GA solution with a 300 g·L^–1^ concentration (black) and an aqueous Carbitol solution with a concentration of 200 g·L^–1^ (red). Carbitol:GA combined in a 2:1 molar ratio (orange). (b) Bulk Raman data for aqueous solutions of Carbitol:GA in a 2:1 molar ratio. There is no change to the carbonyl region, and the carbonyl remains at the Raman wavelength expected for GA (585 nm or 1702.97 cm^–1^). The legend corresponds to days since the reactants were combined.

Figure 5(a) shows an aerosol phase experiment containing a 3:1 molar ratio of Carbitol and GA with water, where the droplet was dried to RH < 5%. This stoichiometry is representative of the Takeda et al. patent used to produce the diester and carbonyl shifts. In contrast to bulk solution experiments where no changes in the Raman spectra were observed over long time periods (Figure 4), for the aerosol droplet experiment the wavelength of the carbonyl peak increased as the RH decreased. The carbonyl Raman shift changes from 1702.97 to 1732.14 cm^–1^ (a wavelength shift from 585 to 586 nm), corresponding to a change in the value very close to that presented in the patent IR data for the carbonyl stretch of dicarbitol succinate (1732.98 cm^–1^). If this signifies the formation of the ester, the reaction appears to occur very quickly on drying, with the peak shifting on a time scale of ∼400 s. Figure 5(b) shows the increase in the relative band integral of the product between 584 and 587 nm.

**Figure 5:**
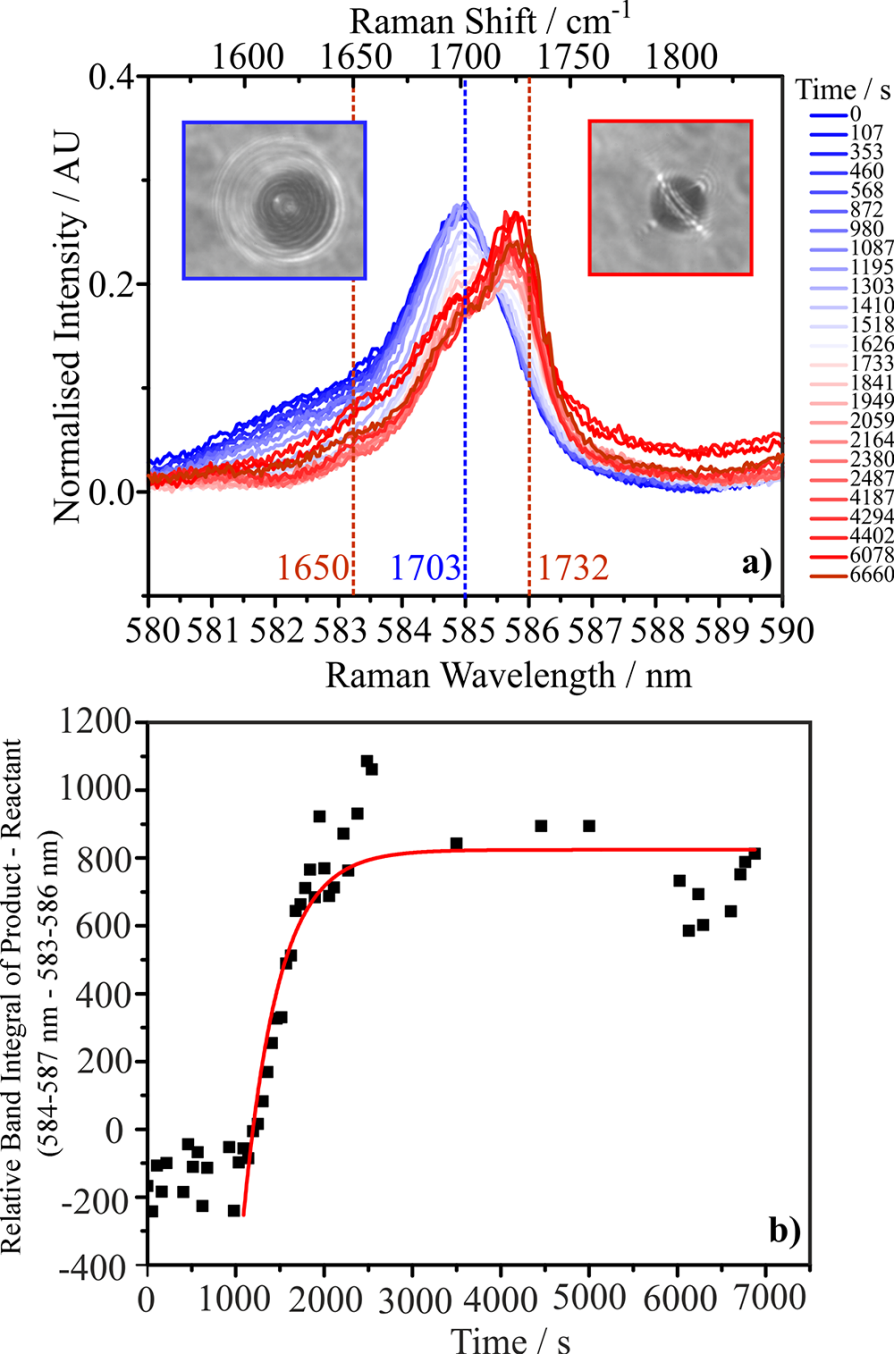
(a) The carbonyl region (580–590 nm) for an aqueous droplet containing Carbitol and GA in a 3 to 1 molar ratio. An increase in the Stoke’s shift of the carbonyl stretch is observed when the RH is lowered from 80 to 0% (blue at time 0 s to red at ∼6500 s). The legend indicates the time after trapping at which the Raman spectrum was acquired. The brightfield imaging shows a droplet before and after RH is dropped. (b) Time-dependence of the difference between the product and reactant band integrals; red line fitted $y=A_{1}e^{-\left(x-x_{0}\right)/t1}+y_{0}$ where *t*_1_ = 396.4 s.

Figure S4 shows dehydration of a droplet containing a 2:1 Carbitol:GA mixture. A similar change in intensity ratio between the 1703.0 to 1732.1 cm^–1^ carbonyl peaks is observed. Moreover, the shape of the carbonyl peak in Figure S4 indicates that some unreacted GA remains in the droplet from the remaining presence of the carbonyl peak at 584.95 nm. In fact, an additional peak at a Raman shift of ∼1650 cm^–1^ suggests that remaining unreacted carboxylic acid groups have formed dimers, which occur at a lower Raman shift as a result of weakening of the carbonyl bond.^65^ Observation of dimer formation is likely due to the lower 2 to 1 stoichiometry of Carbitol to GA.

Intriguingly, the shifting composition on drying appears to be reversible in the droplet phase. Figure 6 shows the repeated dehydration and rehydration cycles for a droplet containing a 3:1 mixture of Carbitol and GA, indicating that the carbonyl peak returns to its original wavelength (585 nm) on hydration. Indeed, these observations suggest that the equilibrium of the reaction is readily controlled by the gas phase RH, leading to ester formation at low RH, and that the reverse reaction can be similarly catalyzed under appropriate conditions, as well as the forward reaction. There are two potential driving factors that could contribute to this observation. One is the increasing water content, which drives the reaction back to reactants. The other is that the reverse reaction may be acid-catalyzed as water adsorbs to the aerosol, just as the forward reaction is acid-catalyzed.

**Figure 6:**
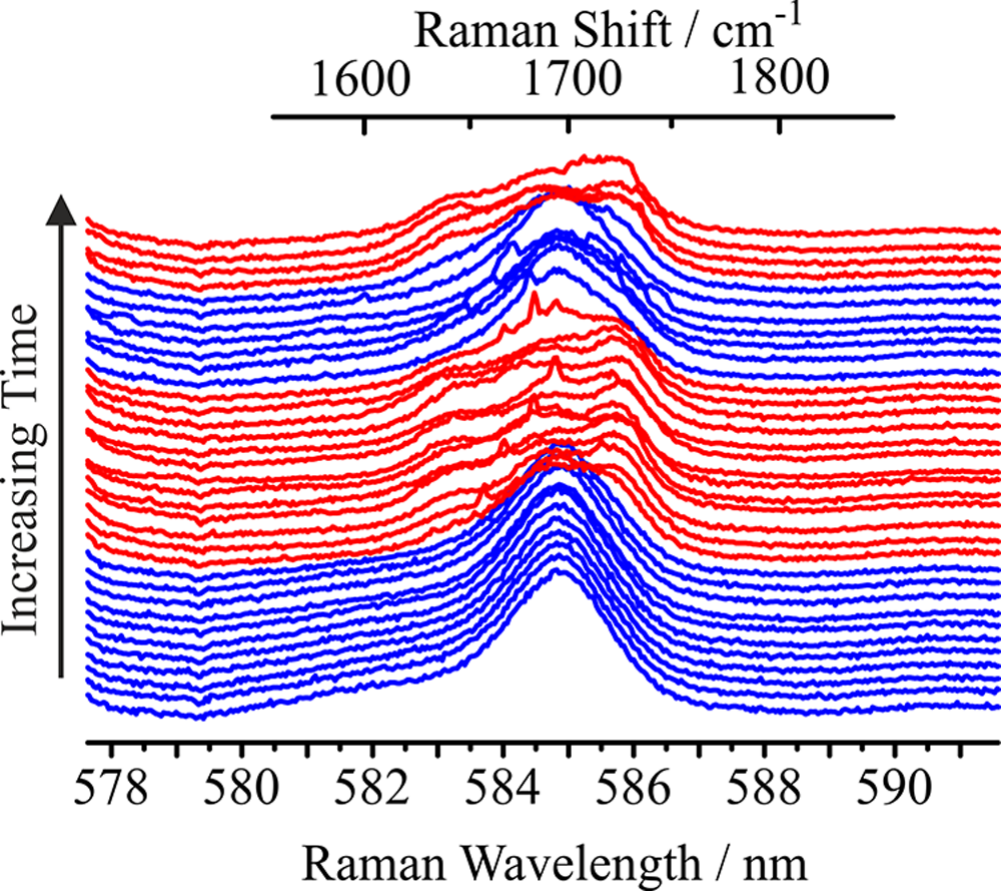
An aqueous droplet containing a solution of Carbitol and GA in a 3:1 molar ratio. At 80% RH (blue) the carbonyl peak is static at 585 nm; on decreasing the RH to 0% (red), the carbonyl peak moves to a higher wavelength of 586 nm. When the RH is raised again, the carbonyl peak returns to its initial value. All spectra have been normalized to the same value and offset for clarity.

### Further Evidence of Ester Formation in Fine Mode Dried Droplets

Esterification during droplet drying was confirmed through measurements made on sub-femtoliter droplets. In one set of experiments, the aerosol processed through the continuous aerosol flow reactor was collected, generating sufficient product for offline analysis by ^1^H NMR. In the other set of experiments, the aerosol was dried and sampled directly into a MS for chemical analysis.

The ^1^H NMR spectrum of a bulk solution of Carbitol and GA is shown in Figure 7 (black line). As expected, the NMR spectrum corresponds to a mixture of the two individual components. NMR spectra for GA and Carbitol in CDCl_3_ are shown for comparison. ^1^H NMR spectra for the solid product extracted after the aerosol flow reactor experiment are also shown in Figure 7 (blue line). After operating the atomizer for ∼45 min, ∼0.0123 g of a white crystal was obtained from the bubbler solution on drying under nitrogen. From Figure 7, the triplet at 4.23 ppm is indicative of protons adjacent to an ester bond. Individual ^1^H NMR spectra (Figure S5) and all ^1^H NMR peaks are reported in the SI along with comparison ^1^H NMR predictions made using ChemDRAW (Section S4, Tables S1–S4). Of relevance is the increase in chemical shift of glutaric acid protons upon esterification.

**Figure 7:**
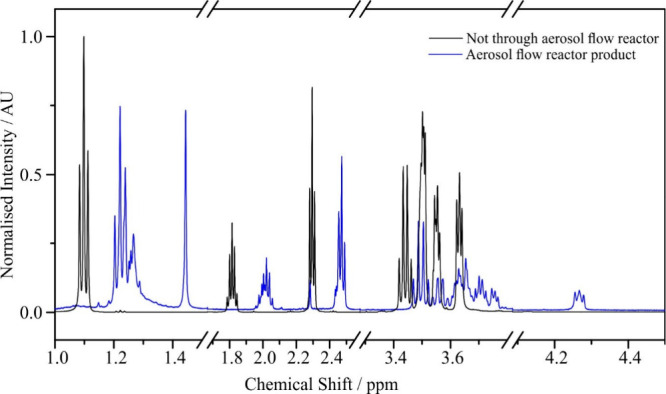
NMR spectra for the bulk solution (black line, not passed through the aerosol flow reactor) and the solution after being passed through the aerosol flow reactor (blue line).

MS experiments were separately conducted on aerosol droplets generated without applied external voltage by atomizing a 2:1 Carbitol:GA aqueous solution, conditioning the aerosol to a desired RH in a Nafion dryer, and then chemically analyzing it by DAI-MS. Example DAI mass spectra for wet (90% RH) and dry (50% RH) droplets are provided in Figure S6. DAI mass spectra often exhibit ions with sodium and potassium adduction.^59,61,66^ Similar ions are observed in both mass spectra, although their relative abundances change depending on the experimental conditions. Ion identities are detailed in Table S5 and were confirmed through comparisons to predicted monoisotopic masses, mass spectra of the reactants individually (Figure S7), and fragmentation patterns observed upon collision-induced dissociation. In the DAI mass spectra, ions associated with the reactants Carbitol (e.g., at 135.1 *m*/*z* and 157.1 *m*/*z*) and glutaric acid (e.g., 155.0 *m*/*z* and 177.0 *m*/*z*) were identified along with ions associated with the monoester products (271.1 *m*/*z* and 287.1 *m*/*z*, corresponding to sodium and potassium adducts, respectively). Additionally, ions at 289.1 *m*/*z* and 305.1 *m*/*z* appear to be sodium and potassium adducted intermediate clusters containing both Carbitol and glutaric acid. Collision-induced dissociation on these ions yields the corresponding monoesters rather than the reactants. The relative abundance of monoester products in the mass spectra was quantified by summing the signal intensities associated with the two monoester ions and then dividing by the sum of all ion intensities (reactants and monoester products) within the mass spectra. The two ions identified as intermediates were not included in this calculation. (Their relative abundance increases slightly with decreasing RH; including them as either reactants or products only marginally changes the observed RH-dependent trends.) MS conditions (e.g., source block temperature, collision voltage) were maintained constant throughout all experiments.

The relative abundances of monoester ions in the mass spectra are plotted as a function of RH in Figure 8. As RH is reduced from ∼90% to ∼40% RH, the relative abundance of the monoester ions increases by approximately an order of magnitude. These observations are consistent with the aerosol flow reactor experiments, where product was observed when the aerosol was dried before deposition and NMR analysis, but minimal product formation was formed within the (wet) bulk solution. Moreover, these results are consistent with the RH-dependent AOT measurements on 10 μm droplets, which show increasing ester formation at lower RH.

**Figure 8:**
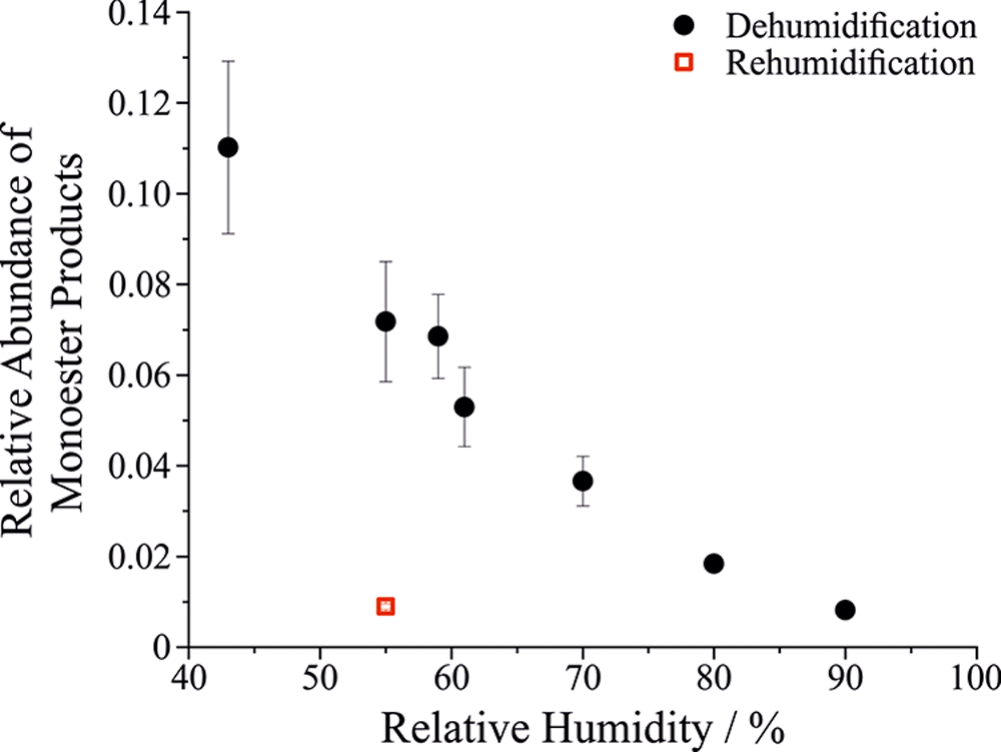
Relative abundance of monoester products as a function of RH. Filled black circles are for data where the aerosol was equilibrated within a Nafion dryer before delivery to the MS. The open red square is for an experiment where the aerosol was equilibrated to 55% RH and then sent to a Condensation Growth Chamber to rehumidify the aerosol before delivery to the MS.

The open red square in Figure 8 demonstrates the reversibility of the esterification process on the nanoscale. In this experiment, the atomized aerosol was dried in the Nafion dryer to 55% humidity (forming some ester, as demonstrated by the black circle at this RH). The RH-conditioned aerosol was then sent to the Condensation Growth Chamber, where it was grown hygroscopically to ∼4 μm diameter before being sampled into the MS. Figure 8 demonstrates that when the aerosol is grown by water condensation before introduction to the MS, the reaction reverses and the observed relative abundance of the monoester is now more similar to that observed at very high humidity. This observation of the reaction’s reversibility on the nanoscale is consistent with the conclusions drawn on the microscale droplet described in Figure 6. The equilibrium of the reaction is readily controlled by the gas phase RH owing to Le Chatelier’s principle. Also notable is that the droplet lifetimes for these experiments are generally <2 s, indicating the reaction is occurring on a similar or shorter time scale on the nanoscale, whereas product formation is observed to take place over hundreds of seconds in the 10 μm droplets.

### Studies of the Esterification Reaction of Succinic, Malonic, Tartaric, and Citric Acids with Carbitol

Aerosols containing additional acids were explored to confirm esterification is observed beyond just the GA:Carbitol system. AOT measurements on droplets containing succinic acid and malonic acid (the analogous C4 and C3 diacids, respectively) show the same trends as GA:Carbitol experiments, suggesting that they also form esters (see Figure S8). By contrast, aerosols containing tartaric and citric acids show no indication of reaction. For tartaric acid and citric acid mixtures, Carbitol appears to evaporate entirely under dry conditions over a time frame of 100,000 s and the RI increases toward that of tartaric acid or citric acid. The evaporative loss of Carbitol from droplets containing mixtures of tartaric or citric acid prevents efflorescence and has been previously used to determine the pure component melt RI of tartaric or citric acid.^64^ The evaporation is clearly shown in Figure 9a, where data are presented for a 3:1 mixture of Carbitol and tartaric acid. Evaporation of Carbitol is observed both in the disappearance of the relevant spontaneous Raman bands (i.e., the loss of the signature between 628 and 629 nm) and in the RI, which tends to that of pure tartaric acid, 1.4966 (Figure 9a).^64^ The RI measured here is considerably higher than the 1.44 value seen in GA/Carbitol mixtures under dry conditions, indicative of a particle that is composed just of the acid. The Raman band also does not show the appearance of a new signature in the carbonyl region indicative of an ester, as shown in Figure 9d.

**Figure 9:**
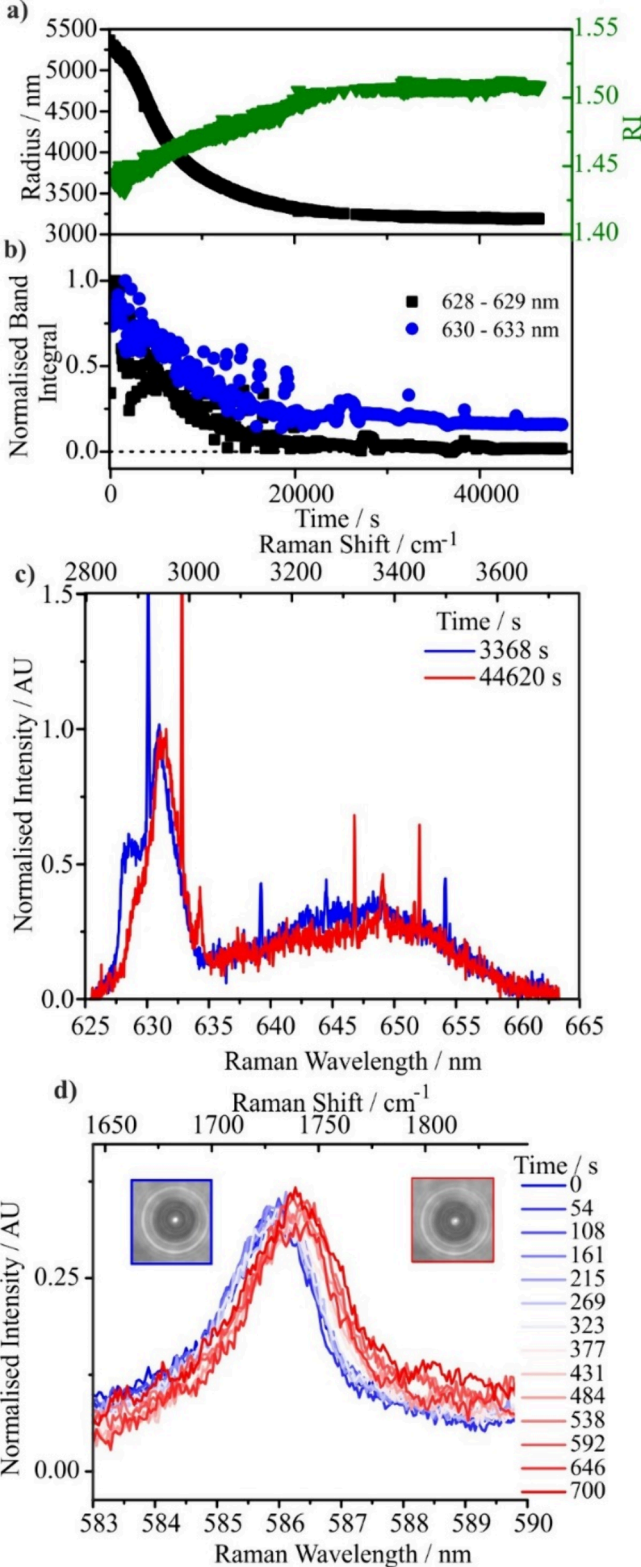
Carbitol and tartaric acid (TA) mixture (3:1), (a) radius and RI vs time; the RI tends to that of pure component TA, where RH is lowered immediately after trapping. Shown in (b) is the band intensity of a signature indicative of Carbitol between 625 and 630 nm during the course of the experiment in (a) (dashed line is the integral of zero). (c) Raman spectra taken at early (blue, 80% RH) and late (red, 0% RH) time from the experiment in (a), the Carbitol signature (625–630 nm). In (d) another experiment with the same mixture as (a), (b), and (c) but with the spectrograph centered at 575 nm and the RH changed from 80% (blue) to 0% (red), to view the carbonyl region; brightfield imaging shows no changes to droplet morphology on drying and very minimal change to the carbonyl peak on dehydration.

These trends with acid identity were confirmed by DAI-MS experiments that explored the same systems as well as 3-methylglutaric acid, 3,3-dimethylglutaric acid, and adipic acid (a C6 analogue). Figure 10 shows the ratio of relative monoester signal intensities in the aerosol at 55% RH and 90% RH conditions. Similar to the AOT studies in 10 μm droplets, relatively more ester product signal was identified in aerosol generated from solutions containing Carbitol and glutaric, 3-methylglutaric, 3,3-dimethylglutaric, succinic, malonic, or adipic acids that was dried to low RH compared to when that aerosol was conditioned to high RH. Moreover, no ions associated with ester products were identified in the mass spectra of the aerosol generated from solutions containing Carbitol and tartaric or citric acids.

**Figure 10:**
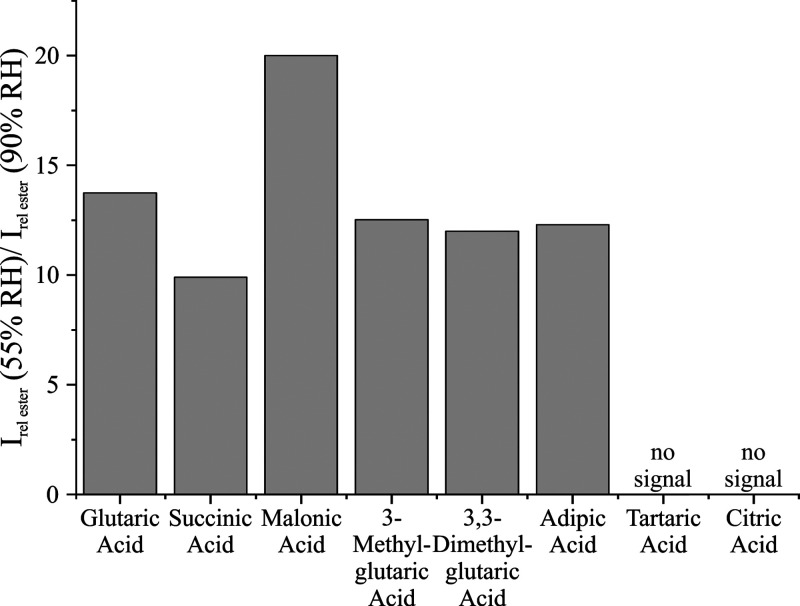
Ratio of the relative DAI-MS signal intensities for the monoester product at 55% RH and 90% RH conditions for aerosols containing different organic acids and Carbitol. No signal intensity was observed in aerosol containing Carbitol and tartaric or citric acids.

The different behavior of the glutaric/succinic/malonic/adipic acid and citric/tartaric acid systems may be influenced by the final viscosity achieved on dehydration and/or the chemical structure of the reacting species. The large increase in viscosity of droplets containing citric acid on drying (and likely tartaric acid given the number of hydroxyl and carboxylic acid groups)^67^ will result in a reduction in diffusivity and slowing of the evaporation rate, as reported for tartaric acid in Figure 9. The viscosity of a droplet containing citric acid increased from ∼10^–1^ at 80% RH to 10^6^ Pa·s under dry conditions. Indeed, the presence of Carbitol (a liquid at room temperature) can be expected to plasticize the particle, thus preventing complete suppression in volatility. By contrast, the viscosity of a glutaric acid containing droplet is unlikely to increase above 1 Pa·s upon drying.^67^ In addition, steric hindrance in the citric/tartaric acid systems with additional functional groups on the bridging alkyl backbone may prevent successful product formation.

## Conclusions

We present evidence for esterification reactions in droplets containing Carbitol and glutaric, succinic, or malonic acid upon droplet drying in picoliter and sub-femtoliter volume droplets. The application of multiple methods including Raman spectroscopy, NMR, and online MS all independently confirm the accelerated reactivity. We find that in droplets containing Carbitol and glutaric acid, dehydration leads to esterification in <400 s in picoliter droplets and in <2 s in sub-femtoliter droplets. These time scales are considerably faster than the patented bulk phase synthesis procedure, which requires reflux at high temperature for ∼16 h. Droplet charge is unlikely to be driving this chemistry. The picoliter droplets studied in the AOT were generated from a nebulizer, and the charge across those droplets is expected to follow a Boltzmann distribution. Multiple optical tweezers experiments, each on a different individual droplet, were conducted, and no dramatic droplet-to-droplet differences in reactivity were observed, suggesting the low amounts of droplet charge on these droplets do not affect the chemistry. By rigorously controlling water activity from dilute to solvent-free (0% RH) conditions, we show a clear dependence of product yield on RH. Intriguingly, the reaction is shown to be reversible with changing water activity, demonstrating the droplets provide a unique environment to catalyze both the forward and backward reactions, and consistent with expectations from Le Chatelier’s principle. Overall, this benchmark system demonstrates the possibility to exploit aerosol reactions for rapid, controlled chemical synthesis.

## Supplementary Material



## Data Availability

Data underlying the figures are available through the University of Bristol data repository, data.bris, at https://doi.org/10.5523/bris.3of4jwnf4avjj2961ostazx9dq.

## Notes

Experimental details and figures for determining the vapor pressure of Carbitol, bulk Raman spectra of Carbitol:GA solutions, dehydration experiment for a droplet containing 2:1 Carbitol:GA, all measured and calculated NMR peaks, droplet-assisted ionization mass spectra, and dehydration experiments for droplets containing succinic and malonic acid mixtures with Carbitol (PDF)
