# Supplementary material for: Enhancement of Esterification Reaction Rates in Solvent-Free Aerosol Droplets
Source: J Am Chem Soc. 2026 Mar 20;148(12):12630–40. doi: 10.1021/jacs.5c18218 (PMC13047690; doi:10.1021/jacs.5c18218)
Supplement: Supplementary file 1 [file ja5c18218_si_001.pdf]

Enhancement of Esterification Reaction Rates in Solvent-Free Aerosol Droplets

Joshua Harrison,[a] Aleksandra Marsh,[a] Rachael E. H. Miles, Allen E. Haddrell, Bryan R. Bzdek,* and Jonathan P. Reid*

School of Chemistry, University of Bristol, Cantock's Close, Bristol, BS8 1TS, United Kingdom

[a]These authors contributed equally to this work.

*Corresponding authors: b.bzdek@bristol.ac.uk, j.p.reid@bristol.ac.uk

SUPPORTING INFORMATION

Section S1: Determination of the Vapour Pressure of Carbitol

An EDB was used to measure evaporation profiles of Carbitol aerosol. The pure component vapour pressure (p_i) of Carbitol was determined to be 10.8 ± 0.13 Pa using Equation S1, where: r is droplet radius, t is time, M_i is molecular weight (134 $g \cdot mol^{-1}$), D_i is the gas phase diffusion constant (7×10^{-6} $m^2 \cdot s^{-1}$), R is the ideal gas constant (8.314 $J \cdot mol^{-1} \cdot K^{-1}$), T is the temperature (293 K) and ρ is the density (0.999 $g \cdot cm^{-3}$).

$$\frac{dr^2}{dt} = -\frac{2M_iD_i}{RT\rho}p_i \qquad \text{(S1)}$$

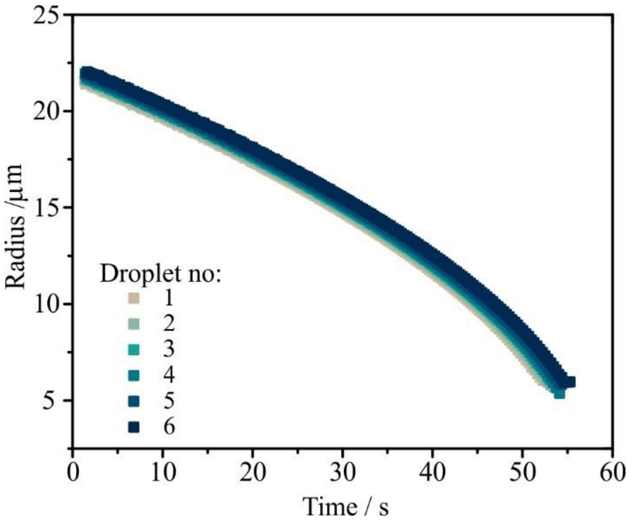

Figure S1: Evaporation profiles of Carbitol aerosol, at 0% RH measured using an EDB.

Section S2: Bulk Raman Spectra of Carbitol:GA Solutions

The measurements presented in Figure S2 (a) – (c) were performed using the aerosol optical tweezers equipment reconfigured to allow for measurements of bulk solutions. A small amount of the bulk solution was pipetted onto the surface of the cover slip and Raman spectra were obtained of the bulk solution using the existing experimental set up.

Figure S2 shows bulk Raman spectra for an aqueous mixture of a Carbitol:GA mixture where the pH of the mixture has been lowered to 0.8 using a 1M solution of HCl.

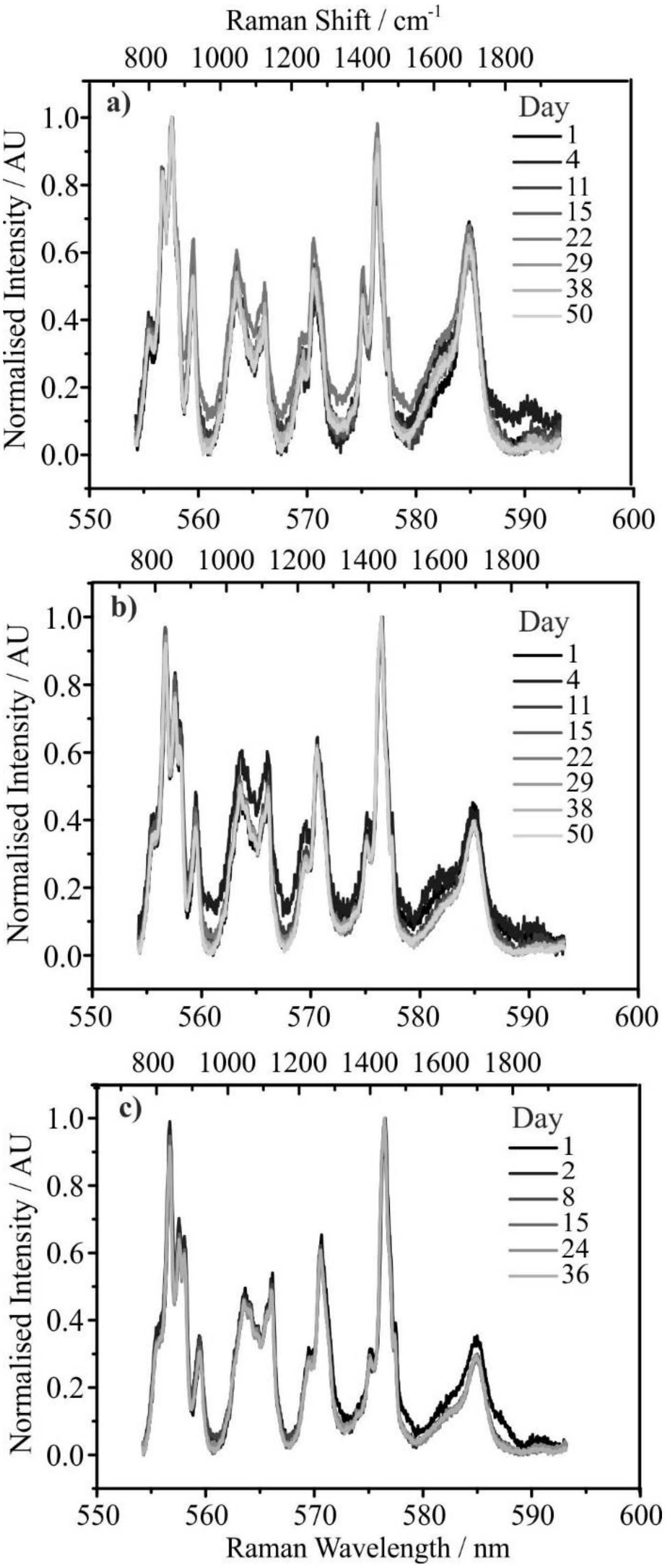

Figure S2: Bulk Raman spectra, taken using the 1200 g·mm^{-1} grating and the spectrograph centred at 575 nm, for three aqueous solutions containing the Carbitol and GA combined in the following molar ratios, 1:1 (a), 2:1 (b) and 3:1 (c). There is no change to the carbonyl region for any of the samples and the carbonyl remains at the Raman wavelength expected for GA (584.95 nm or 1702.97 cm^{-1}). The legend corresponds to the number of days which have passed since the solution was prepared.

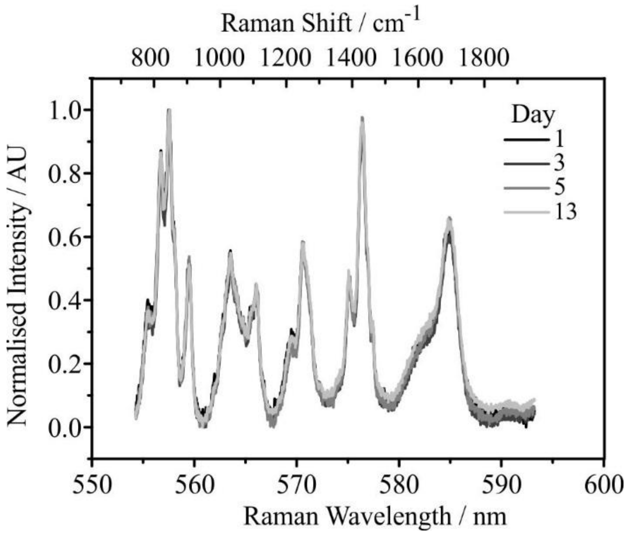

Figure S3: Raman spectra for a 2:1 Carbitol:GA where the pH has been lowered to 0.8 using 1 M HCl. No change to the carbonyl region (580 – 590 nm) is observed and the carbonyl peak (585 nm) corresponds to the carbonyl of GA in all the spectra. The legend corresponds to the date each Raman spectrum was taken.

Section S3: Droplet Dehydration Experiment (Droplet Containing 2:1 Carbitol:GA)

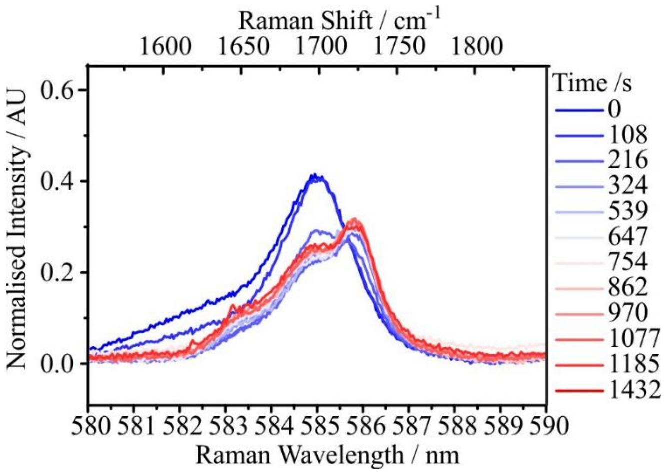

Figure S4: Carbonyl stretch for a dehydrated droplet containing Carbitol and GA combined in a 2:1 molar ratio. An increase in the Stoke's shift of the carbonyl stretch is observed when the RH is lowered from 80 to 0 % (blue to red). However, here there is a distinct change to the peak shape. The legend indicates the time after trapping at which the Raman spectrum was acquired.

Section S4: NMR Reported Peaks

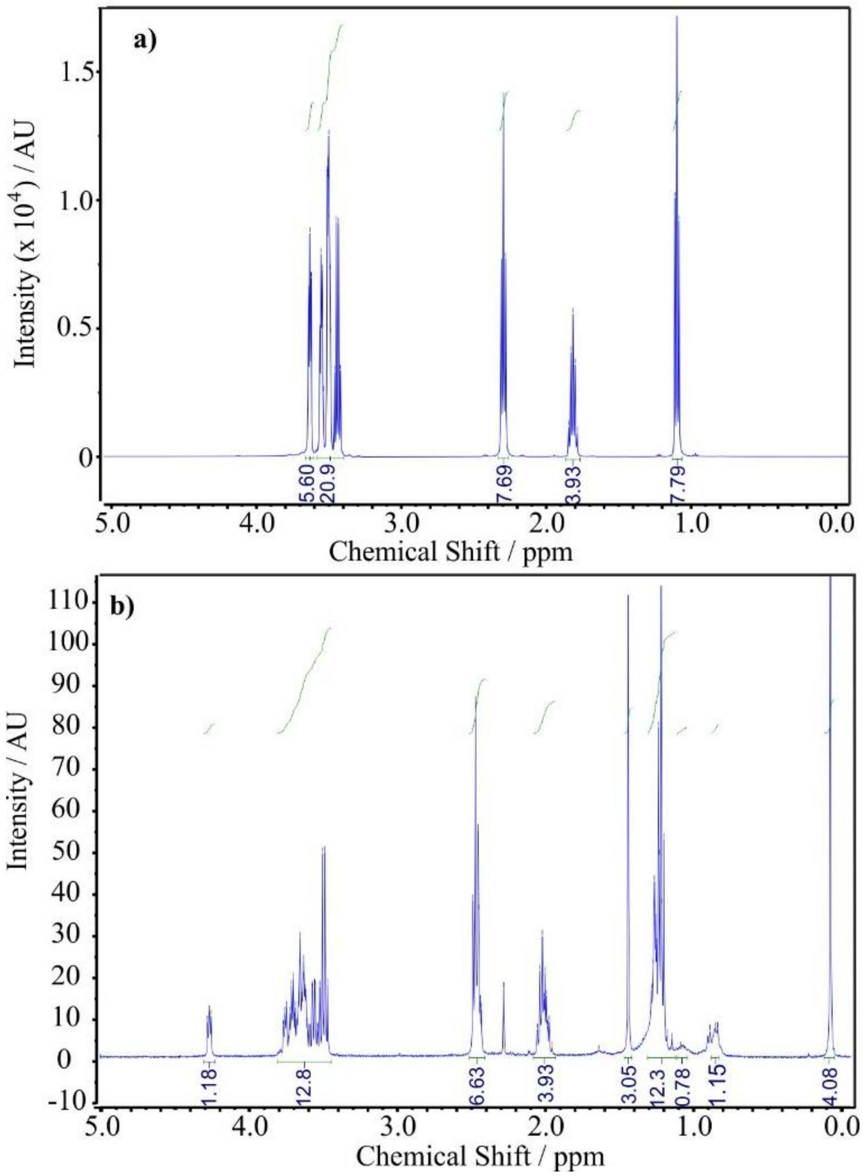

Figure S5: NMR of (a) Bulk solution (not passed through aerosol flow reactor) (b) solutions passed through aerosol flow reactor.

a) GA and Carbitol bulk solution not passed through atomiser: ^{1}H NMR (500 MHz, $CDCl_3$) δ 7.04, 3.64, 3.63, 3.62, 3.56, 3.55, 3.55, 3.54, 3.54, 3.51, 3.51, 3.50, 3.50, 3.49, 3.49, 3.46, 3.45, 3.43, 3.42, 2.31, 2.30, 2.28, 1.84, 1.83, 1.82, 1.80, 1.79, 1.11, 1.10, 1.08.

b) Atomised product: ^{1}H NMR (400 MHz, $CDCl_3$) δ 7.27, 6.99, 5.02, 4.28, 4.27, 4.26, 3.77, 3.76, 3.75, 3.72, 3.71, 3.70, 3.69, 3.69, 3.68, 3.67, 3.66, 3.65, 3.64, 3.64, 3.63, 3.62, 3.61, 3.61, 3.59, 3.59, 3.57, 3.57, 3.56, 3.55, 3.54, 3.54, 3.52, 3.50, 3.49, 3.47, 2.49, 2.47, 2.45, 2.45, 2.44, 2.43, 2.28, 2.06, 2.04, 2.03, 2.02, 2.01, 2.00, 1.99, 1.99, 1.98, 1.96, 1.64, 1.44, 1.29, 1.28, 1.27, 1.26, 1.25, 1.24, 1.23, 1.22, 1.22, 1.20, 1.18, 1.15, 1.09, 0.91, 0.89, 0.86, 0.84, 0.08.

S4.1 NMR Predicted Spectra from ChemDRAW

Table S1: Glutaric acid ^1H NMR Prediction (Lib=SU Solvent=DMSO 300 MHz).

Node (Shift)	Base + Inc.	Comment (ppm rel. to TMS)
OH (12.01)	11	carboxylic
	0	1 -C
	1.01	general
OH (12.01)	11	carboxylic
	0	1 -C
	1.01	general
CH_2 (2.30)	1.37	methylene
	0.9	1 alpha
	-0.06	1 beta
	0.09	general
CH_2 (2.30)	1.37	methylene
	0.9	1 alpha
	-0.06	1 beta
	0.09	general
CH_2 (2.01)	1.37	methylene
	0.23	1 beta
	0.23	1 beta
	0.18	general

Table S2: Carbitol ^1H NMR Prediction (Lib=SU Solvent=DMSO 300 MHz).

Node (Shift)	Base + Inc.	Comment (ppm rel. to TMS)
OH (5.4)	4.2	alcohol
	1.2	1 -CCO
CH_2 (3.52)	1.37	methylene
	2.04	1 alpha -O-C
	0.13	1 beta -O-C
	-0.02	general corrections
CH_2 (3.52)	1.37	methylene
	2.04	1 alpha -O-C
	0.13	1 beta -O-C
	-0.02	general corrections
CH_2 (3.54)	1.37	methylene
	2.04	1 alpha -O-C
	0.15	1 beta -O
	-0.02	general corrections
CH_2 (3.46)	1.37	methylene
	0	1 alpha -C
	2.04	1 alpha -O-C
	0.05	general corrections
CH_2 (3.7)	1.37	methylene
	2.2	1 alpha -O
	0.13	1 beta -O-C
	0	general corrections
CH_3 (1.05)	0.86	methyl
	0.25	1 beta -O-C
	-0.06	general corrections

Table S3: Mono-ester ^{1}H NMR Prediction (Lib=SU Solvent=DMSO 300 MHz).

Node (Shift)	Base + Inc.	Comment (ppm rel. to TMS)
OH (12.01)	11	carboxylic acid
	0	1 -C
	1.01	general corrections
CH_2 (4.20)	1.37	methylene
	2.75	1 alpha -OC(=O)-C
	0.13	1 beta -O-C
	-0.05	general corrections
CH_2 (3.63)	1.37	methylene
	2.04	1 alpha -O-C
	0.24	1 beta -OC(=O)-C
	-0.02	general corrections
CH_2 (3.52)	1.37	methylene
	2.04	1 alpha -O-C
	0.13	1 beta -O-C
	-0.02	general corrections
CH_2 (3.52)	1.37	methylene
	2.04	1 alpha -O-C
	0.13	1 beta -O-C
	-0.02	general corrections
CH_2 (3.46)	1.37	methylene
	0	1 alpha -C
	2.04	1 alpha -O-C
	0.05	general corrections
CH_2 (2.36)	1.37	methylene
	0.92	1 alpha -C(=O)O-C
	-0.06	1 beta -C
	0.13	general corrections
CH_2 (2.30)	1.37	methylene
	0.9	1 alpha -C(=O)O
	-0.06	1 beta -C
	0.09	general corrections
CH_2 (2.13)	1.37	methylene

	0.35	1 beta -C(=O)O-C
	0.23	1 beta -C(=O)O
	0.18	general corrections
CH_3 (1.05)	0.86	methyl
	0.25	1 beta -O-C
	-0.06	general corrections

Table S4: Diester ^1H NMR Prediction (Lib=SU Solvent=DMSO 300 MHz).

Node (Shift)	Base + Inc.	Comment (ppm rel. to TMS)
CH_2 (4.2)	1.37	methylene
	2.75	1 alpha -OC(=O)-C
	0.13	1 beta -O-C
	-0.05	general corrections
CH_2 (4.2)	1.37	methylene
	2.75	1 alpha -OC(=O)-C
	0.13	1 beta -O-C
	-0.05	general corrections
CH_2 (3.63)	1.37	methylene
	2.04	1 alpha -O-C
	0.24	1 beta -OC(=O)-C
	-0.02	general corrections
CH_2 (3.63)	1.37	methylene
	2.04	1 alpha -O-C
	0.24	1 beta -OC(=O)-C
	-0.02	general corrections
CH_2 (3.52)	1.37	methylene
	2.04	1 alpha -O-C
	0.13	1 beta -O-C
	-0.02	general corrections
CH_2 (3.52)	1.37	methylene
	2.04	1 alpha -O-C
	0.13	1 beta -O-C
	-0.02	general corrections
CH_2 (3.52)	1.37	methylene
	2.04	1 alpha -O-C
	0.13	1 beta -O-C
	-0.02	general corrections
CH_2 (3.52)	1.37	methylene
	2.04	1 alpha -O-C
	0.13	1 beta -O-C
	-0.02	general corrections

CH_2 (3.46)	1.37	methylene
	0	1 alpha -C
	2.04	1 alpha -O-C
	0.05	general corrections
CH_2 (2.36)	1.37	methylene
	0.92	1 alpha -C
	-0.06	1 alpha -O-C
	0.13	general corrections
CH_2 (2.36)	1.37	methylene
	0.92	1 alpha -C(=O)O-C
	-0.06	1 beta -C
	0.13	general corrections
CH_2 (2.25)	1.37	methylene
	0.35	1 alpha -C(=O)O-C
	0.35	1 beta -C
	0.18	general corrections
CH_3 (1.05)	0.86	methylene
	0.25	1 beta -C(=O)O-C
	-0.06	1 beta -C(=O)O-C
CH_3 (1.05)	0.86	general corrections
	0.25	methyl
	-0.06	1 beta -O-C

Section S5: Droplet-Assisted Ionization-Mass Spectrometry

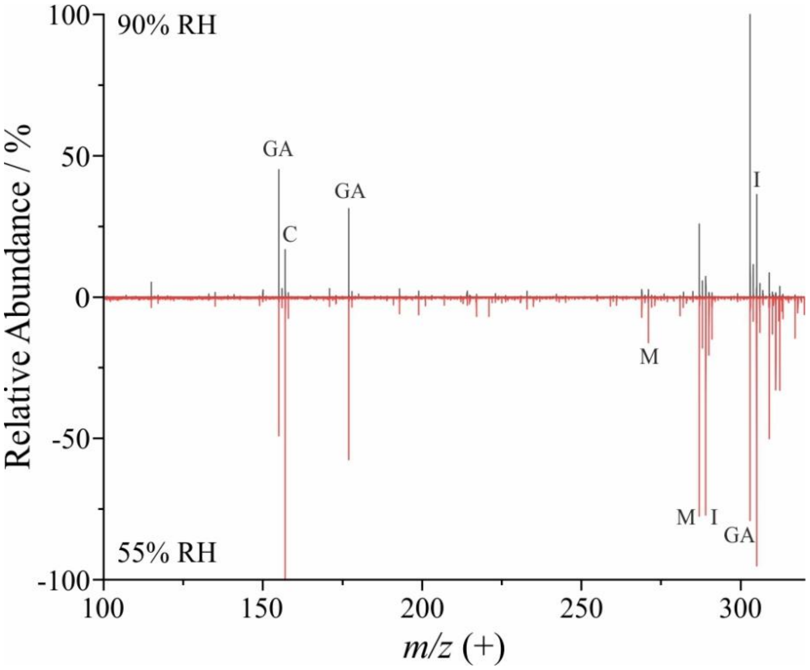

Figure S6: DAI mass spectra of aerosols generated from a solution containing a 2:1 Carbitol:GA mixture at high (top) and low (bottom) RH. Peak assignments are provided in Table S5, but the most intense ions are identified in the mass spectra based on which constituent to which they correspond. Ion identities were determined by comparing the DAI mass spectra with those of the reactants (C and GA) individually, by comparing observed and predicted monoisotopic masses, and by comparing the fragmentation patterns generated using collision-induced dissociation. GA: glutaric acid, C: Carbitol, M: Monoester, I: Intermediate cluster containing Carbitol and glutaric acid**.**

Table S5: Ion assignments in the DAI mass spectra. GA: glutaric acid, C: Carbitol, M: Monoester.

m/z **(+)**	**Assignment**
115.0395	GA-H_2O+H
117.0916	C-H_2O+H
135.0943	C+H
155.0320	GA+Na
157.0841	C+Na
177.0140	GA-H+2Na
271.1158	M+Na
287.0743	2GA+Na
287.0897	M+K
289.1263	C+GA+Na
289.1417	2C-H2O+K
303.0482	2GA+K
305.1003	C+GA+K

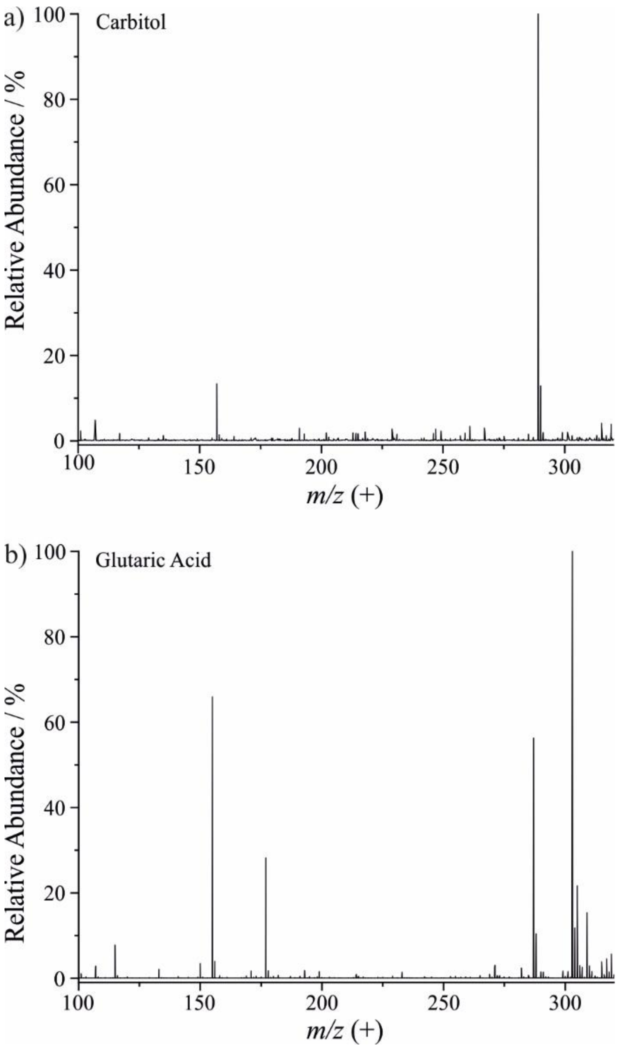

Figure S7: DAI mass spectra of the two reagents individually: a) Carbitol and b) glutaric acid. For Carbitol, prominent peaks are observed at 117.09, 157.08, and 289.14. For glutaric acid, prominent peaks are observed at 115.04, 155.03, 177.01, 287.07, and 303.05 *m/z*. These peaks associated with the individual reactants are consistent with several identified peaks detailed in Fig. S7. Note that although there are is a reagent peak at 287 *m/z* that is nominally isobaric with the sodiated monoester product, these this reagent peak was confidently deconvoluted from the monoester product based on their differing exact masses.

Section S6: Droplet Dehydration Experiments for Aerosol Containing Succinic and Malonic Acid Mixtures with Carbitol

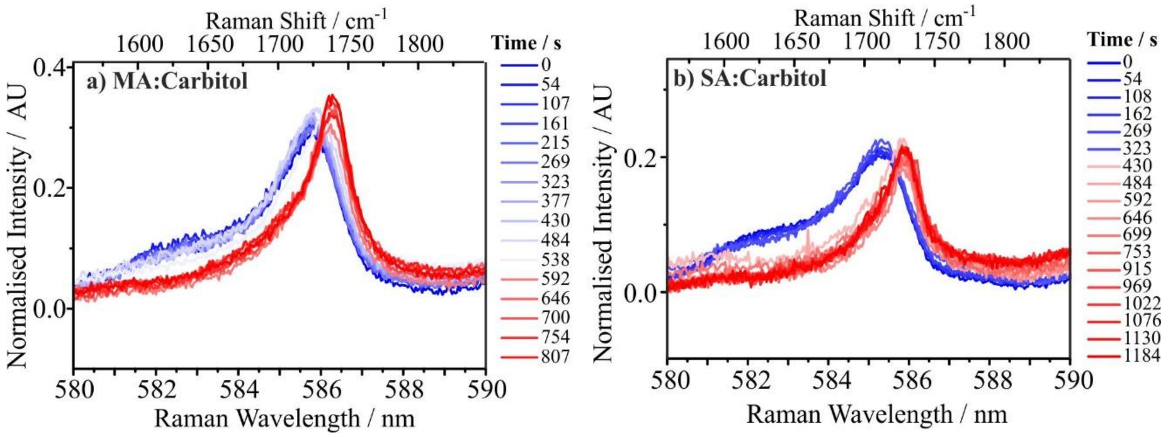

Figure S8: In (a) an aqueous droplet containing MA and Carbitol dried from 80 % RH (blue) to 0 % RH (red) with final RI (0 % RH) of 1.4427. In (b) an aqueous droplet containing SA and Carbitol dried from 80 % RH (blue) to 0 % RH (red) with final RI (0 % RH) of 1.4331.
